# Bat Rabies in Guatemala

**DOI:** 10.1371/journal.pntd.0003070

**Published:** 2014-07-31

**Authors:** James A. Ellison, Amy T. Gilbert, Sergio Recuenco, David Moran, Danilo A. Alvarez, Natalia Kuzmina, Daniel L. Garcia, Leonard F. Peruski, Mary T. Mendonça, Kim A. Lindblade, Charles E. Rupprecht

**Affiliations:** 1 Division of High-Consequence Pathogens and Pathology, Centers for Disease Control and Prevention, Atlanta, Georgia, United States of America; 2 Department of Biological Sciences, Auburn University, Auburn, Alabama, United States of America; 3 United States Department of Agriculture, National Wildlife Research Center, Ft. Collins, Colorado, United States of America; 4 Center for Health Studies, Universidad del Valle de Guatemala, Guatemala City, Guatemala; 5 Centers for Disease Control and Prevention Regional Office for Central America, Guatemala City, Guatemala; 6 Division of Health Protection, Centers for Disease Control and Prevention, Atlanta, Georgia, United States of America; 7 Ross University School of Veterinary Medicine, Basseterre, St. Kitts, West Indies; U.S. Naval Medical Research Unit No. 2, Indonesia

## Abstract

Rabies in bats is considered enzootic throughout the New World, but few comparative data are available for most countries in the region. As part of a larger pathogen detection program, enhanced bat rabies surveillance was conducted in Guatemala, between 2009 and 2011. A total of 672 bats of 31 species were sampled and tested for rabies. The prevalence of rabies virus (RABV) detection among all collected bats was low (0.3%). Viral antigens were detected and infectious virus was isolated from the brains of two common vampire bats (*Desmodus rotundus*). RABV was also isolated from oral swabs, lungs and kidneys of both bats, whereas viral RNA was detected in all of the tissues examined by hemi-nested RT-PCR except for the liver of one bat. Sequencing of the nucleoprotein gene showed that both viruses were 100% identical, whereas sequencing of the glycoprotein gene revealed one non-synonymous substitution (302_T,S_). The two vampire bat RABV isolates in this study were phylogenetically related to viruses associated with vampire bats in the eastern states of Mexico and El Salvador. Additionally, 7% of sera collected from 398 bats demonstrated RABV neutralizing antibody. The proportion of seropositive bats varied significantly across trophic guilds, suggestive of complex intraspecific compartmentalization of RABV perpetuation.

## Author Summary

In this study we provide results of the first active and extensive surveillance effort for rabies virus (RABV) circulation among bats in Guatemala. The survey included multiple geographic areas and multiple species of bats, to assess the broader public and veterinary health risks associated with rabies in bats in Guatemala. RABV was isolated from vampire bats (*Desmodus rotundus*) collected in two different locations in Guatemala. Sequencing of the isolates revealed a closer relationship to Mexican and Central American vampire bat isolates than to South American isolates. The detection of RABV neutralizing antibodies in 11 species, including insectivorous, frugivorous, and sanguivorous bats, demonstrates viral circulation in both hematophagous and non-hematophagous bat species in Guatemala. The presence of bat RABV in rural communities requires new strategies for public health education regarding contact with bats, improved laboratory-based surveillance of animals associated with human exposures, and novel techniques for modern rabies prevention and control. Additionally, healthcare practitioners should emphasize the collection of a detailed medical history, including questions regarding bat exposure, for patients presenting with clinical syndromes compatible with rabies or any clinically diagnosed progressive encephalitis.

## Introduction

Bats (Order: Chiroptera) have been implicated as hosts and reservoirs for numerous emerging infectious diseases, and are considered one of the most relevant groups of mammals in the study of disease ecology [1]. Guatemala is home to some of the world's richest bat biodiversity, with over 104 extant species [2]–[4]. As one representative global disease detection site established by the U.S. Centers for Disease Control and Prevention, enhanced rabies surveillance and pathogen discovery over the past five years targeting bats has facilitated the discovery of numerous novel viral and bacterial agents [5]–[8]. Considering the diversity and zoonotic potential of pathogens detected to date, the most pressing zoonotic threat from bats in Guatemala is *Rabies virus* (RABV), the only *Lyssavirus* documented in the New World [9]. Rabies is defined clinically in humans that present with an acute progressive encephalitis dominated by hyperactivity or paralytic syndromes that eventually deteriorate towards coma and death in nearly 100% of cases [10].

Rabies epizootiology is well appreciated in countries with an established laboratory-based surveillance network. Combined with molecular epidemiology, enhanced and passive surveillance are used to define the geographic distribution of RABV variants, infer the temporal and spatial spread of infections associated with diverse reservoir hosts, identify spillover infections into humans, and to devise relevant prevention and control based upon such information [11]–[13]. Generally, RABV can be divided into two major clades: one comprising variants associated with carnivores around the globe, and another containing variants associated with bats, raccoons and skunks in the New World. In Latin America, RABV is classically recognized as two broadly distinct epizootiological forms or ‘cycles’, one ‘urban’, in which dogs may serve as the primary reservoir host and vector, and the other as a so-called rural or ‘sylvatic’ cycle, involving wildlife [13].

RABV transmitted by vampire bats (*Desmodus rotundus*) represents the most apparent economic and public health threat associated with bats [14]. Vampire bats exist only in Latin America, and range from the Tropic of Cancer in Mexico, to the Tropic of Capricorn in Argentina and Chile [15]. Vampire bats include three monotypic genera, and all three species derive nutrition from feeding on the blood of other vertebrates. A consequence of this unique biological adaptation is that vampire bats are highly effective at transmitting RABV to a wide diversity of mammals, primarily livestock, but also humans, if preferred prey are not widely available [16]. In Latin America, human rabies fatalities have been associated with RABV spillover from frugivorous and insectivorous bats. However, the burden of mortality appears negligible compared to the human rabies burden associated with the common vampire bat [17]–[19].

Laboratory-based surveillance is recommended by the World Health Organization in all RABV enzootic countries. Accurate ongoing prevalence measures are essential in rabies prevention and control, to estimate the burden of disease, to monitor trends to evaluate the effectiveness of case intervention, and to ensure appropriate management of outbreaks [20]. The surveillance system for rabies in Guatemala is passive, where samples from suspected human and animal cases are collected, sent, and tested by the direct fluorescent antibody (DFA) test at one of two laboratories: the Laboratorio Nacional de Salud in Villa Nueva (main facility), or Laboratorio de Ministerio de Agricultura, Ganadería y Alimentación (MAGA) in Quetzaltenango. The number of reported human rabies cases has decreased over the past two decades. Dogs remain the primary RABV vector, and are associated with approximately 73% (44 of 60) of human cases from 1994 to 2012. However, 23% (14 of 60) of these cases were due to an unspecified or unknown exposure [21], [22]. In the U.S., numerous reports link insectivorous bats to the majority of human rabies cases without a history of conventional exposure to RABV [23]. During the same time period in Guatemala, despite detection of over 200 cases of rabies in livestock, and suspected association of such cases with vampire bat rabies, there were no reported cases in bats.

Why RABV has not been detected in Guatemalan bats is unclear, considering RABV has been detected in the majority of bats species tested throughout North America [24]. Very few bats are tested based upon the existing passive surveillance system, and monoclonal antibody or genetic characterization of RABV variants infecting humans, domestic animals and wildlife is not performed. Given the nocturnal and somewhat cryptic nature of bats, transmission dynamics are difficult to study in natural populations, and many critical gaps remain for a basic understanding the epizootiology of different RABV variants maintained in specific bat taxa. In the present study, we report results from field studies conducted in Guatemala from 2009–2011. The objective of the study was to test whether enhanced surveillance would: (1) complement passive surveillance, specifically regarding the presence of RABV in bats, and (2) extend information on the geographic distribution of RABV circulation among bats to assess the public and veterinary health risks associated with bats in Guatemala.

## Methods

### Ethics statement

All animals were captured and handled in accordance with national guidelines (Guide for the Care and Use of Laboratory Animals) [25]. Protocols for animal capture and use were approved by the CDC Animal Care and Use Committee (USA) protocol number 1843 and 2096, and the Animal Care and Use Committee of the Universidad del Valle de Guatemala (Guatemala).

### Enhanced surveillance sampling

**Figure 1:**
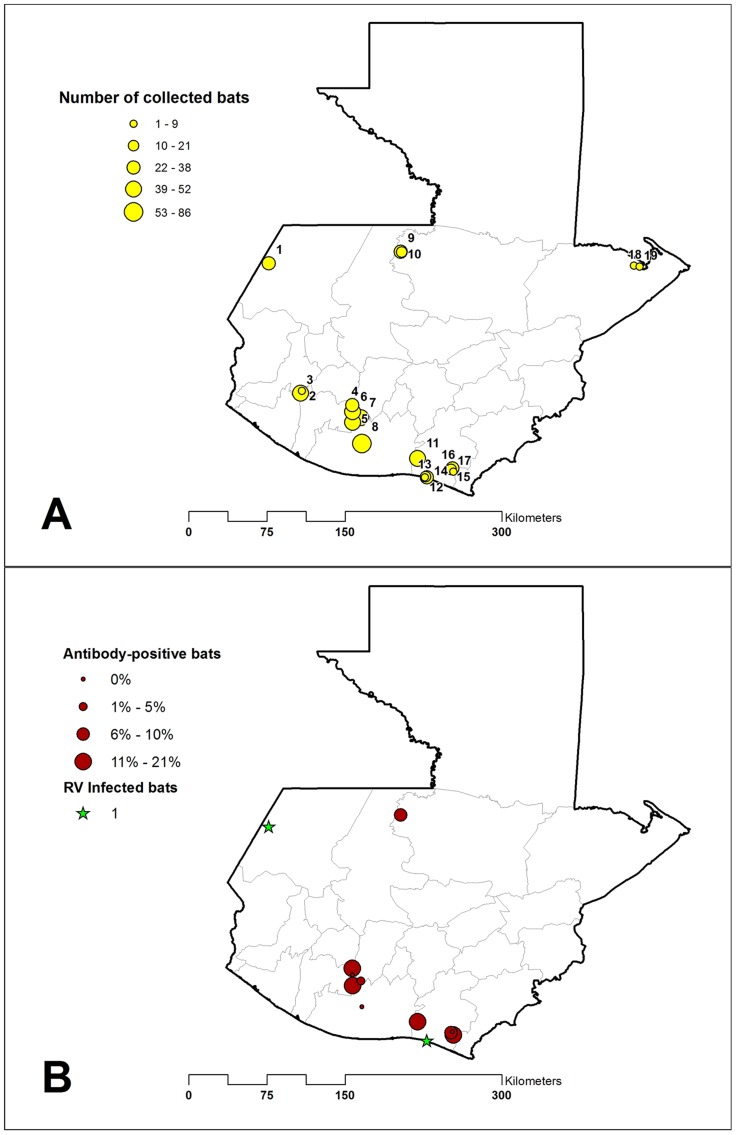
Map of selected field sites, Guatemala 2009–2011. (A) Yellow circles indicate the location 19 sites where bats were tested for RABV antigen and circle size is proportional to the number of bats collected. (B) Red circles indicate the location of ten sites where sera were available for rVNA testing and circle size is proportion to the level of rVNA seroprevalence among tested bats. The location of two rabies infected vampire bats (*D. rotundus*) is indicated by green stars.

Guatemala was selected as one major comparative New World study location as part of the U.S. Centers for Disease Control and Prevention's (CDC) Global Disease Detection (GDD) program among ten international locations. The objective of the CDC GDD program is to develop and strengthen global capacity to rapidly detect, accurately identify, and promptly contain emerging infectious threats. Nineteen field sites for sampling bats in Guatemala were selected on the basis of historical outbreaks of rabies, contemporary national surveillance data, known or suspected vampire bat depredation upon human populations, or neurological illness reported in livestock. Figure 1a illustrates the geographical distribution of field sites used in the present study. Bats were collected using mist nets set near fruit trees, confined livestock, or near the entrance of caves. Nets were opened between 19:00 to 0:00, and checked every 30 minutes. Bats were removed from mist nets, placed individually in cloth bags, and transported to a nearby temporary field station, where they were sedated by a 0.05 to 0.1 mg intramuscular injection of ketamine hydrochloride, oral/fecal swabs obtained, and terminal blood samples were collected by cardiac puncture under heavy anesthesia. Following euthanasia, bats were identified to the level of species following a key for bats of Costa Rica [26]. Standard morphological measurements were also collected (e.g. gender, age, mass, and forearm length). A complete necropsy was then performed on all bats, and samples were stored immediately on dry ice in the field, and maintained thereafter at −70°C in the laboratory at the Universidad del Valle de Guatemala until shipment to the CDC Rabies Laboratory in Atlanta, GA. Carcasses were banded and fixed in 10% buffered formalin for several days, then permanently transferred to 70% ethanol, for archival purposes.

### Direct fluorescent antibody testing

Brain impressions prepared from frozen bat tissues were fixed in acetone at −20°C, and RABV antigens were detected by the DFA test, using fluorescein isothiocyanate (FITC)-labeled monoclonal antibody (mAb) conjugate (Fujirebio Diagnostics, Inc., Malvern, PA, USA), as described [27].

### Serology

The presence of RABV neutralizing antibodies (rVNA) was determined by the rapid fluorescent focus inhibition test (RFFIT) or a modified micro-RFFIT test on sera collected from ten field sites [28], [29]. The rVNA titers of individual bats were calculated by the Reed-Muench method, and were converted to international units (IU/mL) by comparison to a standard rabies immune globulin (SRIG) control containing 2 IU/mL [30]. The SRIG titer was generally higher in the micro-RFFIT test compared to the standard RFFIT. For the objective of this study, positive rVNA titers (≥0.06 IU/mL) were defined by at least 50% neutralization of the RABV challenge virus dose (50 focus forming doses) at a 1∶5 dilution (RFFIT) or 1∶10 dilution (micro-RFFIT). Final titers less than 0.06 IU/mL were considered negative for the presence of rVNA for the purposes of this investigation.

### Antigenic characterization

Antigenic characterization was performed by indirect immunofluorescence using eight mAbs directed against the RABV nucleoprotein (N) antigens (C1, C4, C9, C10, C12, C15, C18, C19), supplied by EMD Millipore Corporation (Billerica, MA, USA), as previously described [31]. Positive reactivity pattern results were analyzed using previous described antigenic variant patterns [32], [33].

### Virus isolation

Virus isolation was attempted on oral and fecal swabs stored in growth medium (MEM-10), and organs, obtained from DFA-positive bats. Homogenates (10% w/v) were prepared in MagnaNA lyser tissue homogenizer tubes containing 1.4-mm (diameter) ceramic beads (Roche Applied Science, Penzberg, Germany), using 1.0 mL of MEM-10 as a diluent. The entire solution from the oral swab, and 0.5 ml from the fecal swab was used to inoculate cells for isolation. For virus recovery, 100 uL of test inoculum was added to 1 mL of MEM-10 containing 5×10^6^ mouse neuroblastoma cells (MNA) in a T-25 tissue culture flask (Corning, NY). Tissue culture flasks were incubated at 0.5% CO_2_ at 37°C for 72 hours. All cultures were sub-passaged a minimum of four times. For infectivity assessments, Teflon-coated four well slides were seeded with 30 uL of MEM-10 containing 0.5×10^6^ cells per mL, and incubated in a humid chamber at 0.5% CO_2_ at 37°C for 24 hours. The slides were then rinsed with phosphate-buffered saline (PBS 4550), and fixed in cold acetone at −20°C for one hour. RABV antigens were visualized by use of the DFA test, using optimal working dilutions of FITC-labeled anti-RABV mAb conjugate (Fujirebio Diagnostics, Inc., Malvern, PA, USA) after each passage.

### RNA extraction, reverse transcription, and PCR

Total RNA was extracted from fecal swabs and organ tissues of RABV-infected bats using TRIZol reagent (Invitrogen, Carlsbad, CA, USA). Fecal swabs were stored in 1 ml of MEM-10 in the field, and 200 µl of the swab suspension was mixed with 1 ml of TRIzol for RNA extraction. Primers were selected within the coding region of the N gene and the initial reaction was performed with sense primer 1066F, GARAGAAGATTCTTCAGRGA (positions 1157–1173), which was also used for reverse transcription and antisense primer 304B, TTGACGAAGATCTTGCTCAT (positions 1514–1533). The hemi-nested reaction was performed with sense primer 1087F, GAGAARGAACTTCARGA (positions 1157–1173). The glycoprotein (G) gene was amplified as two overlapping fragments using primer combinations umf2/994b and 760f/308b, as previously described [34]. All positions are given according to the Street Alabama Dufferin RABV strain genome sequence (GenBank accession number M31046). The RT-PCR was performed as described elsewhere [35]. Positive results were confirmed by nucleotide sequencing. RT-PCR products were purified with Wizard PCR Preps DNA Purification System (Promega, Madison, WI, USA), according to the manufacturer's recommendations and sequenced in forward and reverse directions as described by [35], using the Big Dye Terminator Cycle Sequencing Ready Reaction Kit, version 1.1 on an ABI3770 sequencer (Applied Biosystems, Carlsbad, CA, USA).

### Phylogenetic analysis

The complete N and G sequences were assembled and translated to amino acid sequences using the Bio Edit program [36]. The dataset was supplemented with complete and partial gene sequences available from GenBank and aligned in ClustalX [37]. Table S1 describes details of all sequences used in this study, including accession numbers, country of origin, year, and specimen source. The GTR+I+G model were selected based on the Bayesian factor and Akaike criterion evaluated in MEGA, version 5.1. No molecular clock evaluation was implemented. The analysis was performed using the Bayesian skyline population prior, and two independent Markov Chain Monte Carlo (MCMC) runs were performed with 1,000,000 iterations each. The results were combined in Log Combiner, and the resulting maximum clade credibility (MCC) tree generated with Tree Annotator using 20% for burn in and visualized in Fig Tree, version 1.4.0 [38].

### Statistical analysis

All statistical analyses were performed using JMP version 9.0.2 or SAS version 9.2 (SAS Institute Inc., Cary, NC). The 95% confidence intervals (CI) were calculated for proportions of seropositive bats by species and location of capture. A nested mixed logistic model was used to test the effect of trophic guild on seroprevalence. Species nested within trophic guild was treated as a random effect and trophic guild was treated as a fixed effect. As only a single sanguivorous species was tested, we also compared the model outputs with a data set excluding vampire bats. The antibody prevalence between sex were also compared using χ^2^ tests, and the level of significance was evaluated at α = 0.05.

## Results

### Geographic distribution of bat sampling

During 2009–2011, a total of 672 bats of 31 species were collected from Guatemala. Bats were collected during 2009 (n = 220; Table S1), 2010 (n = 135; Table S2), and 2011 (n = 317; Table S3). Table 1 provides the details on the cumulative frequency of capture by species. Among all captures, 56.3% were male. The most frequently captured species was *D. rotundus* (n = 200; 30%), followed by the Jamaican fruit bat, *Artibeus jamaicensis* (n = 128; 19%) (Table 1).

**Table 1:** Bats collected for rabies testing from 19 field sites in Guatemala, 2009–2011.

Family	Species	Frequency (%)
**Molossidae**		
	*Molossus sinaloae*	2 (0.3)
**Mormoopidae**		
	*Pteronotus davyi*	20 (3.0)
**Noctilionidae**		
	*Noctilio leporinus*	1 (0.1)
**Phyllostomidae**		
	*Artibeus jamaicensis*	128 (19.0)
	*Artibeus lituratus*	35 (5.2)
	*Artibeus phaeotis*	10 (1.5)
	*Artibeus toltecus*	2 (0.3)
	*Carollia brevicauda*	2 (0.3)
	*Carollia castanea*	2 (0.3)
	*Carollia perspicillata*	30 (4.5)
	*Carollia sowelli*	2 (0.3)
	*Centurio senex*	1 (0.1)
	*Chiroderma salvini*	6 (0.9)
	*Chiroderma villosum*	1 (0.1)
	*Desmodus rotundus*	200 (29.8)
	*Glossophaga soricina*	47 (7.0)
	*Lasiurus ega*	1 (0.1)
	*Macrophyllum macrophyllum*	2 (0.3)
	*Micronycteris microtis*	25 (3.7)
	*Phyllostomus discolor*	14 (2.1)
	*Platyrrhinus helleri*	18 (2.7)
	*Sturnira lilium*	96 (14.3)
	*Sturnira ludovici*	2 (0.3)
	*Trachops cirrhosus*	1 (0.1)
	*Uroderma bilobatum*	5 (0.7)
	*Vampyressa pusilla*	2 (0.3)
	*Vampyrodes caraccioli*	1 (0.1)
**Vespertilionidae**		
	*Eptesicus fuscus*	5 (0.7)
	*Myotis elegans*	6 (0.9)
	*Myotis keaysi*	1 (0.1)
	*Myotis nigricans*	4 (0.6)
	**Total**	**672 (100)**

### Serologic evidence of RABV circulation in bats

From sera available for testing (n = 398), 28 bats demonstrated detectable rVNA for an antibody prevalence of 7% (95% CI 5–10%). Seroprevalence was highest for insectivorous species (21%), followed by omnivorous (8%) and sanguivorous (9%) taxa. The proportion of rVNA seropositive bats varied significantly across trophic guilds in a complete data set (F_3,20_ = 4.65, p = 0.04; *Species (Diet) = 3.4×10^−21^*) and a data set without vampire bats (F_2,20_ = 6.97, p = 0.005; *Species (Diet) = 4.4×10^−19^*). In both models, pairwise contrasts revealed a significant difference between frugivorous and insectivorous bats. Insectivorous bats were 8.5 times as likely to be seropositive compared to frugivorous bats. No other pairwise contrasts in rVNA seroprevalence between trophic guild levels were significant. Among capture locations, the proportion of seropositive bats was highest for Naranjo (21%), followed by El Jobo (19%), and El Penate (11%). Table 2 provides the details for individual bat species sampled by location and trophic guild. Antibody prevalence between sexes was similar (3.8% of males, 3.3% of females; *P* = 0.77). Species composition of bats captured varied across sites (Table 2).

**Table 2:** Bat sera tested for rabies virus neutralizing antibodies (positive/tested) from ten collection sites in Guatemala, 2009–2011.

Species	Agüero	El Jobo	El Penate	Finca San Julian	La Viña	Los Hilos	Los Tarrales	Montañas Azules	Naranjo	Salacuim	Subtotal	95% CI
**Frugivorous**											**0.03 (6/199)**	0.01–0.06
*Artibeus jamaicensis*	(0/22)	(0/6)		(0/17)	(1/4)	(0/1)		(0/4)	(0/4)	(0/2)	0.02 (1/60)	0.00–0.09
*Artibeus lituratus*	(0/2)		(1/1)	(0/3)			(0/2)	(0/3)		(0/4)	0.07 (1/15)	0.01–0.30
*Artibeus phaeotis*								(0/1)			0.0 (0/1)	-
*Artibeus toltecus*	(0/1)						(0/1)				0.0 (0/2)	-
*Carollia castanea*							(0/1)		(0/1)		0.0 (0/2)	-
*Carollia perspicillata*	(0/6)	(1/11)	(0/1)	(0/3)		(0/3)	(0/3)	(1/2)			0.07 (2/29)	0.02–0.22
*Centurio senex*										(0/1)	0.0 (0/1)	-
*Chiroderma salvini*				(0/1)							0.0 (0/1)	-
*Platyrrhinus helleri*	(0/1)			(0/2)			(0/2)	(0/10)			0.0 (0/15)	
*Sturnira lilium*	(0/15)	(1/4)	(0/5)	(0/9)	(0/3)	(0/2)	(0/9)	(0/14)	(1/1)	(0/4)	0.03 (2/66)	0.01–0.10
*Sturnira ludovici*					(0/1)		(0/1)				0.0 (0/2)	-
*Uroderma bilobatum*	(0/1)			(0/2)							0.0 (0/3)	-
**Insectivorous**											**0.21 (8/38)**	0.11–0.36
*Vampyressa pusilla*										(0/2)	0.0 (0/2)	-
*Eptesicus fuscus*	(0/3)							(0/1)			0.0 (0/4)	-
*Macrophyllum macrophyllum*	(0/2)										0.0 (0/2)	-
*Molossus sinaloae*	(0/2)										0.0 (0/2)	-
*Myotis elegans*		(0/1)							(1/5)		0.17 (1/6)	0.03–0.56
*Myotis keaysi*								(0/1)			0.0 (0/1)	-
*Myotis nigricans*	(0/1)	(1/1)						(0/1)			0.33 (1/3)	0.06–0.79
*Pteronotus davyi*						(0/1)			(6/19)		0.30 (6/20)	0.15–0.52
**Omnivorous**											**0.08 (6/73)**	0.04–0.17
*Glossophaga soricina*	(0/8)	(2/6)		(0/1)	(0/1)	(0/1)	(1/5)	(0/8)	(0/5)	(0/2)	0.08 (3/37)	0.03–0.21
*Micronycteris microtis*		(1/3)				(0/22)					0.04 (1/25)	0.01–0.20
*Phyllostomus discolor*							(2/10)				0.20 (2/10)	0.06–0.51
*Trachops cirrhosus*										(0/1)	0.0 (0/1)	-
**Sanguivorous**												
*Desmodus rotundus*	(0/22)	(2/10)	(0/2)	(0/5)	(0/6)		(1/4)	(0/5)	(2/13)	(3/21)	**0.09 (8/88)**	0.05–0.17
**Total**	0.0 (0/86)	0.19 (8/42)	0.11 (1/9)	0.0 (0/43)	0.07 (1/15)	0.0 (0/30)	0.11 (4/38)	0.02 (1/50)	0.21 (10/48)	0.08 (3/37)	**0.07 (28/398)**	0.05–0.10
95% CI	-	0.10–033	0.02–0.44	-	0.01–0.30	-	0.04–0.24	0.00–0.10	0.12–0.34	0.03–0.21		

### Detection of RABV and antigenic typing

RABV antigens were detected in the brain from two common vampire bats. The bats were collected in El Pumpo, near the Pacific Coast in the Department of Santa Rosa, and Palo Seco, near the border with Mexico in the Department of San Marcos (Figure 1b). The antigenic reaction pattern derived from the brain specimens were consistent with RABV antigenic variant V3 associated with *Desmodus rotundus* (Table 3). Upon capture, one of the two rabid bats demonstrated clinical signs consistent with rabies infection. Additionally, this bat was dehydrated, in poor physical condition, and had evidence of several bite wounds to its body.

**Table 3:** Antigenic patterns of two bat rabies virus isolates from Guatemala.

	Patterns of reaction (N-mABs)								Antigenic Variant
	C1	C4	C9	C10	C12	C15	C18	C19	
Bat 258	−	+	+	+	+	−	−	+	*Desmodus rotundus* (V3)
Bat 321	−	+	+	+	+	−	−	+	*Desmodus rotundus* (V3)

### Isolation of RABV and detection of viral RNA by hemi-nested reverse transcription-PCR

RABV was isolated in MNA cells 24 h after inoculation from the kidney of one bat, and from oral swabs of both rabid bats. Additional sub-passages revealed the presence of RABV in the spleen of one bat, and the kidney and lung for both bats. RABV was not isolated from the heart, liver, intestine, and fecal swabs. Viral RNA was detected by hemi-nested RT-PCR in all specimens examined, except the liver of Bat 321. The results of RABV isolation and nucleic acid detection from tissues of rabid bats are presented in Table 4.

**Table 4:** Cell culture isolation and primary/hemi-nested (nRT-PCR) results from rabid bat specimens collected in Guatemala.

	Bat 258						Bat 321					
	Virus Isolation				RNA detection^1^		Virus Isolation				RNA detection	
Specimen Source	Passage 1	Passage 2	Passage 3	Passage 4	Primary	Hemi-nested	Passage 1	Passage 2	Passage 3	Passage 4	Primary	Hemi-nested
Fecal swab	−	−	−	−	−	+	−	−	−	−	−	+
Kidney	+	+	+	+	+	+	−	+	+	+	−	+
Spleen	−	−	+	+	+	+	−	−	−	−	−	+
Heart	−	−	−	−	−	+	−	−	−	−	−	+
Liver	−	−	−	−	+	+	−	−	−	−	−	−
Lung	−	+	+	+	−	+	−	+	+	+	+	+
Intestine	−	−	−	−	+	+	−	−	−	−	−	+
Oral Swab	+	+	+	+	NT^2^	NT	+	+	+	+	NT	NT

Sequences from positive RNA detection in this table were not published in Genbank.

1 Primary (primers 1066:304), Hemi-nested (primers 1087:304).

2 NT, not tested.

### GenBank accession numbers

In total, two RABV G sequences and two RABV N sequences were generated from the brain tissues of infected bats in this study and deposited into GenBank (accession numbers: KF656696-99).

### Molecular relationships between RABV associated with vampire bats in Latin America

**Figure 2:**
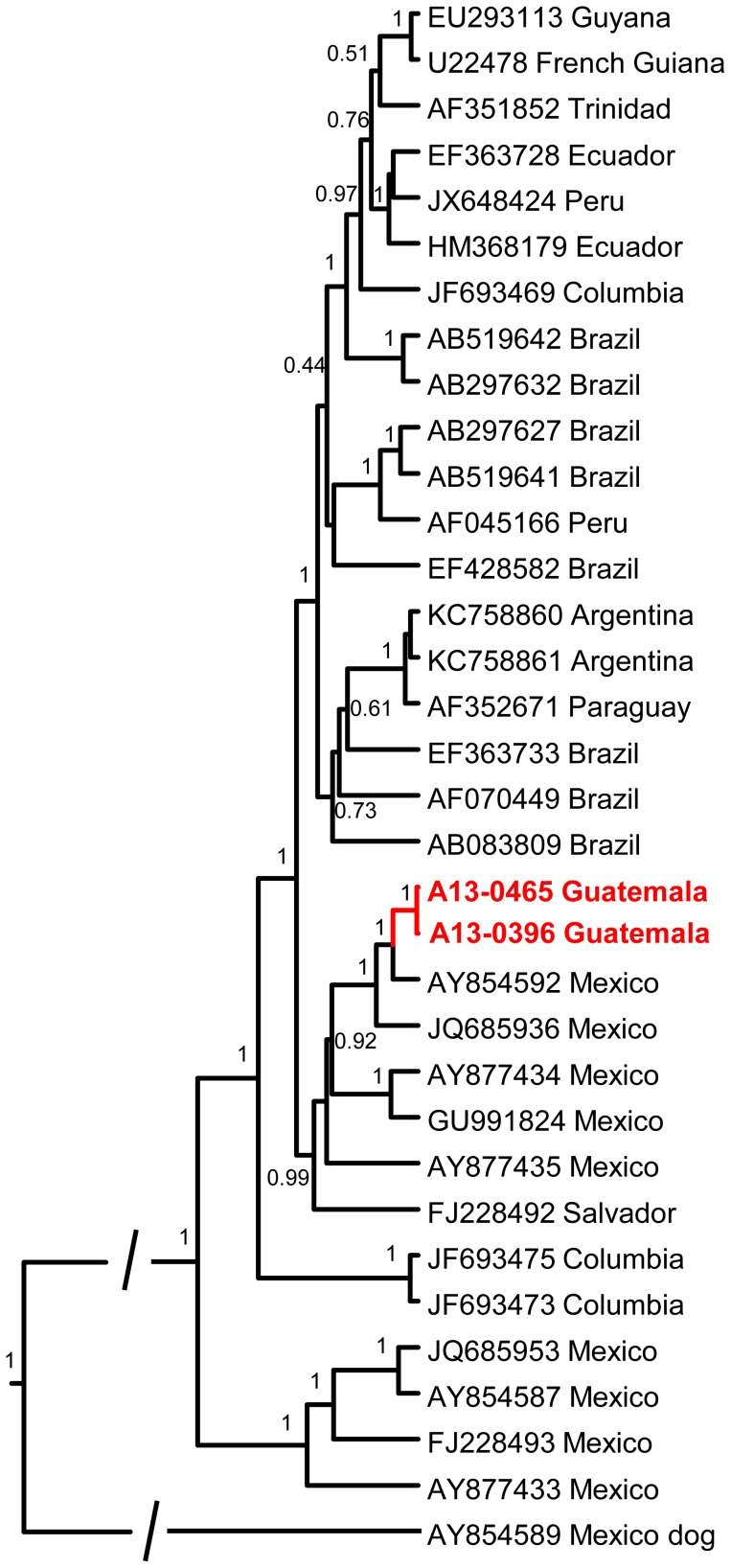
Maximum clade credibility tree of rabies viruses based on complete nucleoprotein (N) gene sequences. Posterior probabilities are shown for key nodes. Mexico dog RABV was used as an out-group. Novel sequences generated from the CNS material of two rabid vampire bats in this study are shown in red.

The N gene sequences of both viruses were 100% identical, whereas the G sequences differed in one non-synonymous substitution (302_T,S_). The viruses were most similar to one of the lineages of vampire bat rabies viruses circulating in Mexico, and were relatively distant from the lineages circulating in South America, with the exception of Columbian lineages. The most phylogenetically related viruses were described previously from Chiapas, Tabasco and Veracruz states of eastern Mexico [39]. The only available vampire bat RABV N gene sequence from El-Salvador (FJ228492) also clustered within this lineage (Figure 2, G gene reconstruction not shown).

## Discussion

Given the critical ecological importance of bats, especially in tropical regions, novel strategies are necessary for the prevention and control of bat-associated zoonoses. To our knowledge, this is the first report and isolation of RABV from bats in Guatemala. In this study, the prevalence of rabies among all collected bats was low (0.3%), even among vampire bats (1%). The two vampire bat RABV isolated were related phylogenetically to viruses associated with vampire bats in the eastern states of Mexico and El Salvador, which is not unexpected given their geographic proximity. Rabies epizootics and phylogenetic clusters of RABV circulating in vampire bat populations are relatively constrained in space [40], [41]. Previous phylogeographic studies suggested that vampire bat rabies likely originated in the territory of Mexico [39]. If true, these viruses may have been introduced to Guatemala and El Salvador via eastern Mexico. The detection of rVNA from bats in this study demonstrated RABV exposure among multiple species of bats in Guatemala. The overall rVNA prevalence of 7% was similar to other bat RABV surveillance studies conducted in Peru (10.3%), Grenada (7.6%) and Trinidad (12.8%), but less than the 37% detection in Colima, Mexico [42]–[44]. Seroprevalence among collection sites ranged from 0 to 21% among all bats, and 0 to 25% for *D. rotundus*, respectively. Our results are concordant with previous studies in demonstrating that a substantial fraction of apparently healthy bats have detectible rVNA, indicating previous exposure to RABV, and suggesting clearance of peripheral infection without clinical disease [42]–[44]. The presence of rVNA only demonstrates prior exposure to RABV antigens, and does not provide information about the timing, intensity, or frequency of infection [45]. Furthermore, rVNA negative test results do not guarantee a lack of exposure, and may vary according to the selected cutoff value used for the serology test, as reviewed by Gilbert et al. [46]. At a population level, the rVNA seroprevalence data described here provide information about the cumulative exposure history among all bats collected for each field site.

In experimentally infected bats, rVNA are typically generated only among survivors, and bats that succumb to infection may only seroconvert during late stages of clinical disease [45]. Similar observations have been made regarding rVNA seroconversion in human rabies patients [47]. Combined with antigen detection results, rVNA seroprevalence data can be used to elucidate infection dynamics in bat populations. Detection of RABV excretion can provide meaningful insights for identifying potential transmission pathways that can inform the structure of disease dynamic models. In this study, the highest levels of virus were consistently detected from oral swabs, kidney and lung tissue. These results support primary secretion by oral routes, while suggesting a possible pathway in urine (via the kidneys), which warrants further investigation.

Strategies to control RABV transmitted by vampire bats in Latin America have relied historically on population reduction methods, either by non-specific destruction of roosts or by anti-coagulants applied to cattle or individual bats. When the anti-coagulant paste is applied to vampire bats, it is suspected that the treated bats then return to a roost where is spread to other conspecifics through allogrooming. Culling of vampire bats is thought to benefit agriculture and public health in the short-term by alleviating bat bites on livestock and humans respectively. However, there is little evidence that this method actually targets rabid bats, and the apparent positive effect of culling on seroprevalence, combined with demographic and behavioral responses, may actually increase the proportion of susceptible bats [41], [48]. A recent report also suggests that it may be more economically beneficial to support pre-exposure vaccination of cattle, than rely on vampire bat culling [49].

Rabies surveillance at the national level in Guatemala falls under the jurisdiction of the Ministry of Public Health and Social Welfare [50]. Rabies control efforts in Guatemala are focused primarily on mass vaccination of domestic dogs, which has led to a significant decrease in human rabies cases, though it remains unclear whether current efforts can achieve ultimate elimination of canine rabies [21], [51]. Indirect vaccination of vampire bats with recombinant RABV vaccines have proven immunogenic and efficacious in experimental infection models of *D. rotundus*, though this strategy has not been tested in the field [52]. Despite possibly reducing RABV infection of cattle, this strategy would not eliminate the behavior or health and economic consequences (e.g. secondary bacterial infections) of vampire bat depredation on cattle. Interventions at the human-bat interface should be directed at decreasing the risk of human exposure to bats by improved educational campaigns on the risk of rabies associated with bats and the importance of laboratory testing and prophylactic treatment following exposures, as well as bat-proofing dwellings where feasible [53]. Routine laboratory-based RABV surveillance is necessary to properly evaluate any intervention strategy and should include the submission and testing of all human and animal cases involving bite contact with bats and other reservoir hosts. For Guatemala, overcoming cold chain and transportation barriers should be a priority to increase the proportion of contact cases that are tested. To further elucidate the relative impacts of the urban or sylvatic cycles on public health and agriculture, routine typing of all positive rabies cases should be implemented. Rapid exchange of information between sectors involved in human and animal rabies surveillance and control is essential.

Unfortunately, despite significant progress in the prevention and control of rabies, once clinical signs manifest in humans the case fatality rate approaches 100%, even with intensive supportive care [47]. However, appropriate post-exposure prophylaxis, including immediate washing/flushing and disinfection of the wound and prompt administration of RIG, and modern cell-culture vaccines according to recommended vaccination schedules assures prevention of rabies if bitten by a rabid bat [54]. Fortunately, modern cell culture vaccines are readily available for both humans and animals in Guatemala, and national authorities should provide pre-exposure prophylaxis guidelines for persons or animals with regular exposure to bats or other potential wildlife reservoirs (e.g. carnivores).

As the first focused rabies study in Guatemala, gaps were evident in our study. Limitations to the current study included a lack of more focal seasonal sampling, a broader spatial scale, and a need for greater engagement of the Ministries of Health and Agriculture in a One Health context to improve routine laboratory based surveillance. The presence of bat RABV in rural communities likely requires new strategies for joint public health and veterinary education and outreach, increased availability for diagnostic laboratory testing in remote areas, and continued enhanced surveillance for rabies prevention and control, as suggested for other developing countries [55], [56]. Additional studies at the human-bat interface would be useful to obtain information on demographic characteristics (e.g. age, gender, education) of persons exposed to bats, circumstances of bat exposures (e.g. bites, scratches, skin contact), actions taken following exposure, and knowledge of bat-borne zoonoses. Previously, based upon ignorance or inaction, preventable human rabies cases have occurred from bat exposure [57]–[59]. Given the implications when such recommendations are not operative, localities with a high risk for exposure to bats, and bat-borne zoonoses, should be targeted in laboratory based surveillance activities for the evaluation of robust, long-term prevention and control strategies.

## Supporting Information

Table S1 Bats collected for rabies testing from eight field sites in Guatemala, 2009.
(DOCX)

Table S2 Bats collected for rabies testing from two field sites in Guatemala, 2010.
(DOCX)

Table S3 Bats collected for rabies testing from nine field sites in Guatemala, 2011.
(DOCX)

Table S4 List of sequences included in the data set from Central and South American vampire bat RABV.
(DOCX)
