# Supplementary material for: Bat Rabies in Guatemala
Source: PLoS Negl Trop Dis. 2014 Jul 31;8(7):e3070. doi: 10.1371/journal.pntd.0003070 (PMC4117473; doi:10.1371/journal.pntd.0003070)
Supplement: Table S1 — Bats collected for rabies testing from eight field sites in Guatemala, 2009. (DOCX) [file pntd.0003070.s001.docx]

**Table S1**. Bats collected for rabies testing from eight field sites in Guatemala, 2009.

| Species | **El Jobo** | **El Penate** | **La Viña** | **Los Hilos** | | **Los Tarrales** | | **Montañas Azules** | **Naranjo** | **Salacuim** | Subtotal |
| --- | --- | --- | --- | --- | --- | --- | --- | --- | --- | --- | --- |
| *Artibeus jamaicensis* | 6 |  | 4 | | 1 | |  |  | 4 | 2 | 17 |
| *Artibeus lituratus* |  | 1 |  | |  | | 2 |  |  | 4 | 7 |
| *Artibeus toltecus* |  |  |  | |  | | 1 |  |  |  | 1 |
| *Carollia castanea* |  |  |  | |  | | 1 |  | 1 |  | 2 |
| *Carollia perspicillata* | 11 | 1 |  | | 3 | | 3 |  |  |  | 18 |
| *Centurio senex* |  |  |  | |  | |  |  |  | 1 | 1 |
| *Desmodus rotundus* | 10 | 2 | 6 | |  | | 4 |  | 13 | 21 | 56 |
| *Glossophaga soricina* | 6 |  | 1 | | 1 | | 5 |  | 5 | 2 | 20 |
| *Micronycteris microtis* | 3 |  |  | | 22 | |  |  |  |  | 25 |
| *Myotis elegans* | 1 |  |  | |  | |  |  | 5 |  | 6 |
| *Myotis nigricans* | 1 |  |  | |  | |  |  |  |  | 1 |
| *Phyllostomus discolor* |  |  |  | |  | | 10 |  |  |  | 10 |
| *Platyrrhinus helleri* |  |  |  | |  | | 2 |  |  |  | 2 |
| *Pteronotus davyi* |  |  |  | | 1 | |  |  | 19 |  | 20 |
| *Sturnira lilium* | 4 | 5 | 3 | | 2 | | 9 | 1 | 1 | 4 | 29 |
| *Sturnira ludovici* |  |  | 1 | |  | | 1 |  |  |  | 2 |
| *Trachops cirrhosus* |  |  |  | |  | |  |  |  | 1 | 1 |
| *Vampyressa pusilla* |  |  |  | |  | |  |  |  | 2 | 2 |
| **Total** | 42 | 9 | 15 | | 30 | | 38 | 1 | 48 | 37 | **220** |
