# Supplementary material for: Bat Rabies in Guatemala
Source: PLoS Negl Trop Dis. 2014 Jul 31;8(7):e3070. doi: 10.1371/journal.pntd.0003070 (PMC4117473; doi:10.1371/journal.pntd.0003070)
Supplement: Table S2 — Bats collected for rabies testing from two field sites in Guatemala, 2010. (DOCX) [file pntd.0003070.s002.docx]

**Table S2**. Bats collected for rabies testing from two field sites in Guatemala, 2010.

| Species | **Agüero** | **Montañas Azules** | Subtotal |
| --- | --- | --- | --- |
| *Artibeus jamaicensis* | 22 | 4 | 26 |
| *Artibeus lituratus* | 2 | 3 | 5 |
| *Artibeus phaeotis* |  | 1 | 1 |
| *Artibeus toltecus* | 1 |  | 1 |
| *Carollia perspicillata* | 6 | 2 | 8 |
| *Desmodus rotundus* | 22 | 5 | 27 |
| *Eptesicus fuscus* | 3 | 1 | 4 |
| *Glossophaga soricina* | 8 | 8 | 16 |
| *Macrophyllum macrophyllum* | 2 |  | 2 |
| *Molossus sinaloae* | 2 |  | 2 |
| *Myotis keaysi* |  | 1 | 1 |
| *Myotis nigricans* | 1 | 1 | 2 |
| *Platyrrhinus helleri* | 1 | 10 | 11 |
| *Sturnira lilium* | 15 | 13 | 28 |
| *Uroderma bilobatum* | 1 |  | 1 |
| **Total** | 86 | 49 | **135** |
