# Supplementary material for: Bat Rabies in Guatemala
Source: PLoS Negl Trop Dis. 2014 Jul 31;8(7):e3070. doi: 10.1371/journal.pntd.0003070 (PMC4117473; doi:10.1371/journal.pntd.0003070)
Supplement: Table S3 — Bats collected for rabies testing from nine field sites in Guatemala, 2011. (DOCX) [file pntd.0003070.s003.docx]

**Table S3**. Bats collected for rabies testing from nine field sites in Guatemala, 2011.

| Species | **Don Israel** | **Don Neto, El Pumpo** | **Finca Don Hugo** | **Finca Guadalupe** | **Finca Las Conchas** | **Finca Las Pavas** | **Finca San Julian** | **Palo Seco** | **Finca El Pumpo** | Subtotal |
| --- | --- | --- | --- | --- | --- | --- | --- | --- | --- | --- |
| *Artibeus jamaicensis* |  | 12 | 2 | 10 | 5 | 1 | 17 | 18 | 20 | 85 |
| *Artibeus lituratus* | 3 | 2 | 2 | 6 |  |  | 3 | 1 | 6 | 23 |
| *Artibeus phaeotis* |  | 2 |  | 3 |  | 1 |  |  | 3 | 9 |
| *Carollia brevicauda* |  |  |  | 2 |  |  |  |  |  | 2 |
| *Carollia perspicillata* |  |  |  |  | 1 |  | 3 |  |  | 4 |
| *Carollia sowelli* |  | 2 |  |  |  |  |  |  |  | 2 |
| *Chiroderma salvini* |  | 1 | 3 | 1 |  |  | 1 |  |  | 6 |
| *Chiroderma villosum* |  |  |  | 1 |  |  |  |  |  | 1 |
| *Desmodus rotundus* |  | 3 | 24 | 30 | 1 | 24 | 5 | 10 | 20 | 117 |
| *Eptesicus fuscus* |  |  |  |  |  | 1 |  |  |  | 1 |
| *Glossophaga soricina* | 1 | 2 | 1 | 3 |  |  | 1 |  | 3 | 11 |
| *Lasiurus ega* |  |  |  |  |  |  |  |  | 1 | 1 |
| *Myotis nigricans* |  |  |  |  |  |  |  |  | 1 | 1 |
| *Noctilio leporinus* |  |  |  |  |  |  |  |  | 1 | 1 |
| *Phyllostomus discolor* |  | 4 |  |  |  |  |  |  |  | 4 |
| *Platyrrhinus helleri* |  |  | 1 | 1 |  |  | 2 |  | 1 | 5 |
| *Sturnira lilium* |  | 8 | 9 | 11 |  |  | 9 |  | 2 | 39 |
| *Uroderma bilobatum* |  |  |  | 1 |  | 1 | 2 |  |  | 4 |
| *Vampyrodes caraccioli* |  |  |  | 1 |  |  |  |  |  | 1 |
| **Total** | 4 | 36 | 42 | 70 | 7 | 28 | 43 | 29 | 52 | **317** |
