# Supplementary material for: Bat Rabies in Guatemala
Source: PLoS Negl Trop Dis. 2014 Jul 31;8(7):e3070. doi: 10.1371/journal.pntd.0003070 (PMC4117473; doi:10.1371/journal.pntd.0003070)
Supplement: Table S4 — List of sequences included in the data set from Central and South American vampire bat RABV. (DOCX) [file pntd.0003070.s004.docx]

**TableS4.** List of nucleoprotein sequences included in the data set from Central and South American vampire bat RABV.

| **Sequence ID** | **Country** | **Year** | **Specimen Source** |  |
| --- | --- | --- | --- | --- |
| A13-0396 (KF656697) | Guatemala | 2012 | Vampire bat |  |
| A13-0465 (KF656696) | Guatemala | 2012 | Vampire bat |  |
| AB083809 | Brazil | 1998 | Cattle |  |
| AB297627 | Brazil | 2002 | Neotropical fruit bat |  |
| AB297632 | Brazil | 2002 | Vampire bat |  |
| AB519641 | Brazil | 1998 | Great fruit-eating bat |  |
| AB519642 | Brazil | 2000 | Vampire bat |  |
| AF045166 | Peru | 1996 | Human |  |
| AF070449 | Brazil | unknown | Vampire bat |  |
| AF351852 | Trinidad | 1995 | Vampire bat |  |
| AF352671 | Paraguay | 1994 | Vampire bat |  |
| AY854587 | Mexico | 1996 | Vampire bat |  |
| AY854589 | Mexico | 1990 | Dog |  |
| AY854592 | Mexico | 2000 | Cow |  |
| AY877433 | Mexico | 1990 | Cow |  |
| AY877434 | Mexico | 1995 | Horse |  |
| AY877435 | Mexico | 1993 | Cow |  |
| EF363728 | Ecuador | 2005 | Human |  |
| EF363733 | Brazil | 2005 | Human |  |
| EF428582 | Brazil | 2006 | Vampire bat |  |
| EU293113 | Guyana | 1990 | Dog |  |
| FJ228492 | Salvador | 2002 | Human |  |
| FJ228493 | Mexico | 2003 | Cow |  |
| GU991824 | Mexico | 2002 | Vampire bat |  |
| HM368179 | Ecuador | 2007 | Cow |  |
| JF693469 | Columbia | 2002 | Horse |  |
| JF693473 | Columbia | 1996 | Cat |  |
| JF693475 | Columbia | 1995 | Human |  |
| JQ685936 | Mexico | 2009 | Vampire bat |  |
| JQ685953 | Mexico | 2009 | Vampire bat |  |
| JX648424 | Peru | 2002 | Horse |  |
| KC758860 | Argentina | 1996 | Cow |  |
| KC758861 | Argentina | 2001 | Cow |  |
| U22478 | French Guiana | 1994 | Dog |  |
